# Acute Megakaryoblastic Leukemia with t(1;22) Mimicking Neuroblastoma in an Infant

**DOI:** 10.4274/tjh.2013.0189

**Published:** 2015-02-15

**Authors:** Müge Gökçe, Selin Aytaç, Şule Ünal, İlhan Altan, Fatma Gümrük, Mualla Çetin

**Affiliations:** 1 Hacettepe University Faculty of Medicine, Division of Pediatric Hematology, Ankara, Turkey

**Keywords:** Acute megakaryoblastic leukemia, t(1;22), Acute myeloid leukemia

## Abstract

Acute megakaryoblastic leukemia (AMKL) with t(1;22) (p13;q13) is an extremely rare subtype of acute myeloid leukemia that is almost always described in infants. t(1;22) (p13;q13)-positive AMKL with extramedullary infiltration has been previously reported only once in the literature. Herein, we report a 3-month-old infant presenting with a pelvic mass and pancytopenia suggesting neuroblastoma. Bone marrow evaluation revealed t(1;22)-positive AMKL that responded well to a regimen containing high-dose cytarabine.

## Abstract (tr)

t(1;22) (p13;q13) pozitif akut megakaryoblastik lösemi (AMKL) olgularının neredeyse hepsi sadece süt çocukluğu döneminde bildirilmiş olup, akut miyeloid löseminin son derece nadir bir alt tipidir. Bu olgu sunumunda, pelvik kitle ve pansitopeni ile başvuran ve tanı anında nöroblastomu taklit eden üç aylık bebek sunulmaktadır. Hastanın kemik iliği incelemesi ile t(1;22) pozitif AMKL tanısı konulmuş ve yüksek doz sitarabin içeren tedavi rejimine hasta iyi cevap vermiştir.

## INTRODUCTION

Acute megakaryoblastic leukemia (AMKL) is a rare but heterogeneous subtype of acute myeloid leukemia (AML) with diverse morphological and cytogenetic features. Being more common in children than in adults, it constitutes 4%-20% of pediatric AML cases with increasing rates during infancy [1,2,3]. AMKL predominates in children with Down syndrome and is associated with somatic GATA1 mutations [4]. Baruchel et al. first reported the nonrandom association between t(1;22) (p13;q13) and infant AMKL [5]. Just after that, the fusion gene OTT-MAL was identified in patients with t(1;22) [6].

Herein, we report a 3-month-old infant who presented with a pelvic mass and pancytopenia suggesting neuroblastoma, who was diagnosed with AMKL with t(1;22) based on a detailed work-up.

## CASE PRESENTATION

A 3-month-old girl was referred to our hospital due to hepatosplenomegaly and pancytopenia, which were noticed during admission, with the complaints of irritability and intractable crying for 1 week. She was the first child of a healthy couple. The prenatal and natal histories were unremarkable.

She was irritable and pale. Her weight and height were 5 kg (in the 25th percentile according to age) and 59 cm (in the 50th percentile according to age), respectively. No ecchymoses or petechiae were noted. Cardiovascular and respiratory system evaluations were normal. The abdomen was distended and the liver and spleen were palpable 3 and 2 cm below the costal margins, respectively. A firm mass of a diameter of 2x3 cm was palpable in the right lower quadrant.

**Figure 1:**
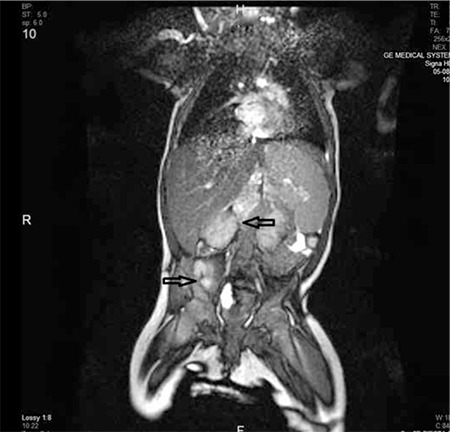
Abdominal magnetic resonance imaging (MRI) with solid mass behind the right psoas major muscle.

Complete blood count revealed hemoglobin of 6.8 g dL-1, platelet count of 8x109 L-1, and leukocyte count of 12.9x109 L-1, with a differential count of 65% lymphocytes, 33% blasts, 1% metamyelocytes, and 1% neutrophils. Serum urea-creatinine and liver function tests were all normal. The lactate dehydrogenase level was 2294 IU L-1. Abdominal ultrasonography yielded multiple hypoechogenic nodules in the liver, packed lymph nodes in the portal area, and a solid mass behind the right psoas major muscle. Abdominal magnetic resonance imaging (MRI) confirmed the ultrasonographic findings (Figure 1). With the preliminary diagnosis of neuroblastoma or hepatoblastoma, serum alpha-fetoprotein (AFP) and urine vanillylmandelic acid (VMA) were analyzed. AFP was 64 IU L-1 (range: 0.5-5.5 IU L-1) and VMA was 36.4 mg g creatinine-1 (normal value: <27 for <12 months of age).

**Figure 2:**
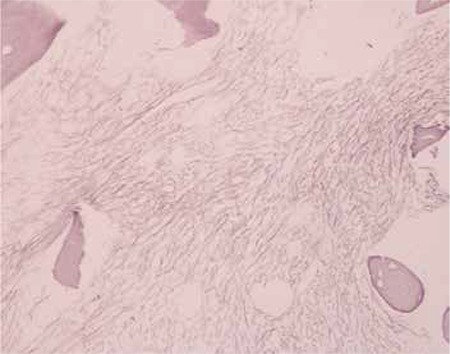
Bone marrow biopsy with increase in reticulin fibers and fibrosis.

Bone marrow aspirate showed overt hypocellularity and scarce myeloblasts with cytoplasmic blebbing without rosette formation. Bone marrow biopsy exhibited an increase in the reticulin fibers and fibrosis (Figure 2). Additionally, CD3, CD20, TdT, CD1a, and AE1-AE3 were negative and CD68, CD34, and S100 were positive in a few cells, while neuron-specific enolase, chromogranin A, and PGP 9.5 were lightly positive in a few cells and synaptophysin was negative. Flow cytometric analysis demonstrated a blast gate of 51% with positivity of CD13, CD33, CD117, and CD42 but negativity of MPO. Conventional cytogenetic evaluation from bone marrow aspirate demonstrated 34-45, XX, t(1;22) (p13;q13) [14]/45,XX.

Evaluating both the clinical presentation and the results of the bone marrow analysis, the diagnosis was AMKL (AML FAB M7). No blasts were detected in the cerebrospinal fluid. An AML BFM 2004 protocol regimen containing high-dose cytarabine was started [7]. Abdominal ultrasonography performed 6 weeks after the initiation of chemotherapy yielded no mass, neither in the liver nor in the neighborhood of the psoas major muscle. She had no matched sibling or unrelated donor. She recently completed therapy and is in complete remission at the second year after diagnosis.

## DISCUSSION AND REVIEW OF THE LITERATURE

AMKL constitutes 4.1%-15.3% of pediatric AML cases in large collaborative studies [8,9,10], and prominent hepatosplenomegaly and bone marrow fibrosis are the characteristic features of this subtype [11]. AMKL in patients with Down syndrome and without Down syndrome are 2 major subgroups of AML. Down syndrome patients with somatic mutations in GATA1 have favorable prognosis, with 90% remission rates and 60%-70% event-free survival [12]. However, AMKL patients without Down syndrome have been reported to have different cytogenetics and poor prognosis. Presence of t(1;22) (p13;q13) cytogenetic anomaly is as low as <1% among all AML patients and 70% of these patients present in the first year of life [13]. The overexpression of the fusion oncogene (OTT-MAL) in the presence of t(1;22) leads to NOTCH signaling deregulation with c-mpl activation [14]. On the other hand, it has been speculated that this protooncogene may also modulate chromatin organization and HOX differentiation pathways. Additional cytogenetic anomalies and/or hyperdiploid clones have been exhibited in 60% of patients [15]. Its prognosis has been thought of as poor, but recent data reported long-term survivors after intensive chemotherapy [16].

In previous reports, the frequency of extramedullary involvement and granulocytic tumors showed wide ranges of 12%-49% and 7%-18%, respectively [3,17,18]. In a previous report from our center, 40% of 127 children with AML were found to have extramedullary infiltration (EMI) at diagnosis and, in this series, FAB M2 and M4+M5 subtypes constituted 31% and 25% of all cases with EMI. None of the patients with EMI were found to have M7 morphology [3].

Presentation with granulocytic sarcoma in AMKL with t(1;22) (p13;q13) has been reported only in a 7-month-old infant until now [13]. Due to the paranasal location of the granulocytic sarcoma in that case, she was misdiagnosed with Burkitt lymphoma, but bone marrow aspiration revealed the diagnosis of AML M7. In the present case, the solid mass behind the right psoas major muscle mimicking neuroblastoma disappeared just after the first cycle of intensive acute nonlymphocytic leukemia-directed therapy and was defined as EMI or granulocytic sarcoma.

To our knowledge, this is the second infant with t(1,22) (p13;q13)-positive AMKL presenting with extensive extramedullary involvement that disappeared immediately after chemotherapy. It should be kept in mind that AMKL with t(1,22) (p13;q13) might mimic solid tumors such as neuroblastoma and patients should be thoroughly investigated for bone marrow involvement. Patients who are unresponsive to other regimens and have atypical presentations of solid tumors must be evaluated for the presence of EMI of AML.
